# Adolescent binge ethanol-induced loss of basal forebrain cholinergic neurons and neuroimmune activation are prevented by exercise and indomethacin

**DOI:** 10.1371/journal.pone.0204500

**Published:** 2018-10-08

**Authors:** Ryan P. Vetreno, Fulton T. Crews

**Affiliations:** The Bowles Center for Alcohol Studies, School of Medicine, University of North Carolina at Chapel Hill, Chapel Hill, North Carolina, United States of America; Universitatsklinikum Leipzig, GERMANY

## Abstract

Basal forebrain cholinergic neurons mature in adolescence coinciding with development of adult cognitive function. Preclinical studies using the rodent model of adolescent intermittent ethanol (AIE; 5.0 g/kg, i.g., 2-days on/2-days off from postnatal day [P]25 to P55) reveal persistent increases of brain neuroimmune genes that are associated with cognitive dysfunction. Adolescent intermittent ethanol exposure also reduces basal forebrain expression of choline acetyltransferase (ChAT), an enzyme critical for acetylcholine synthesis in cholinergic neurons similar to findings in the post-mortem human alcoholic basal forebrain. We report here that AIE decreases basal forebrain ChAT+IR neurons in both adult female and male Wistar rats following early or late adolescent ethanol exposure. In addition, we find reductions in ChAT+IR somal size as well as the expression of the high-affinity nerve growth factor (NGF) receptor tropomyosin receptor kinase A (TrkA) and the low-affinity NGF receptor p75^NTR^, both of which are expressed on cholinergic neurons. The decrease in cholinergic neuron marker expression was accompanied by increased phosphorylation of NF-κB p65 (pNF-κB p65) consistent with increased neuroimmune signaling. Voluntary wheel running from P24 to P80 prevented AIE-induced cholinergic neuron shrinkage and loss of cholinergic neuron markers (i.e., ChAT, TrkA, and p75^NTR^) as well as the increase of pNF-κB p65 in the adult basal forebrain. Administration of the anti-inflammatory drug indomethacin (4.0 mg/kg, i.p prior to each ethanol exposure) during AIE also prevented the loss of basal forebrain cholinergic markers and the concomitant increase of pNF-κB p65. In contrast, treatment with the proinflammatory immune activator lipopolysaccharide (1.0 mg/kg, i.p. on P70) caused a loss of cholinergic neuron markers that was paralleled by increased pNF-κB p65 in the basal forebrain. These novel findings are consistent with AIE causing lasting activation of the neuroimmune system that contributes to the persistent loss of basal forebrain cholinergic neurons in adulthood.

## Introduction

Adolescence is a conserved neurodevelopmental period characterized by significant refinement of neurotransmitter systems that parallels the transition of the immature brain to the more efficient adult brain [1]. The basal forebrain cholinergic system, which is the primary source of acetylcholine innervation to the cortex and hippocampus [2], plays a crucial role in cognitive functioning [3, 4]. While cholinergic neurons are generated early in embryonic development [5–8], these neurons undergo maturational refinement during adolescence [9, 10] that is accompanied by consolidation of cholinergic projections [11–13]. In humans, adolescence is also associated with high levels of alcohol binge drinking [14, 15], which can negatively impact the maturing basal forebrain cholinergic system. Indeed, preclinical rat studies find that adolescent basal forebrain cholinergic neurons are particularly sensitive to ethanol-induced neurodegeneration [16]. Adolescent intermittent ethanol (AIE), which models human adolescent binge drinking, causes a loss of cholinergic neurons immediately following the conclusion of AIE treatment that persists well into adulthood (i.e., P220) [16–20]. Studies comparing our adolescent intermittent ethanol exposure model to an identical adult intermittent ethanol exposure model reveal that adolescents are uniquely sensitive to ChAT+ neuron loss [16] whereas adult loss of ChAT may require months of continuous ethanol exposure [21]. Loss of adult ChAT+ neurons following AIE has been shown to correlate with disruption of novel object recognition memory [20]. The heightened vulnerability of the adolescent brain, coupled with the importance of acetylcholine in cognitive functioning, underscores the importance of identifying the mechanism underlying the persistent loss of basal forebrain cholinergic neurons following adolescent binge ethanol exposure.

While the mechanism underlying the reduction of cholinergic neuron markers in the AIE model [16–19] and human alcoholism [16] remain to be fully elucidated, converging lines of evidence implicate neuroimmune system activation in the loss of basal forebrain cholinergic neurons. Stimulation of the neuroimmune system with the inflammagen lipopolysaccharide (LPS) reduced expression of the cholinergic neuron marker choline acetyltransferase (ChAT), which is the enzyme responsible for acetylcholine biosynthesis, in the rat basal forebrain [22, 23] as well as in cultured cholinergic neurons [24]. Further, basal forebrain infusion of the proinflammatory cytokine TNFα, which is a target gene of the neuroimmune transcription factor nuclear factor kappa-light-chain-enhancer of activated B cells (NF-κB), decreased ChAT+IR neurons [25]. In senescent rats (i.e., ~24–30 months of age), nuclear expression of NF-κB p65 is increased in the basal forebrain accompanying the age-associated reduction of ChAT+IR cholinergic neurons [26, 27]. Similarly, populations of ChAT+IR neurons in the human basal forebrain are diminished in Alzheimer’s disease, which is associated with increased expression of NF-κB p65 [28, 29]. Adolescent binge ethanol exposure has also been shown to increase phosphorylation of NF-κB p65 and induce several proinflammatory NF-κB target genes, including *MCP-1* and *TNF*α throughout the adult brain [30–33]. Together, these data suggest that AIE induction of neuroimmune signaling may contribute to the persistent loss of cholinergic neurons in the adult basal forebrain.

Emerging studies reveal that upregulation of neuroimmune signaling by ethanol is associated with induction of multiple neuroimmune signaling molecules, including proinflammatory cytokines and their receptors, oxidases, and other neuroimmune genes [31–35], most of which converge on NF-κB [36]. The large number of neuroimmune signaling molecules induced by ethanol creates uncertainty in efforts to select specific targets for therapeutic intervention. Interestingly, aerobic exercise has been found to blunt neuroimmune activation in the basal forebrain of rats following middle cerebral artery occlusion [37] and restore basal forebrain cholinergic neurons in a preclinical rodent model of thiamine deficiency-induced cholinergic neurodegeneration [38]. Thus, we tested the hypothesis that voluntary wheel running exposure from postnatal day (P)24 –P80 would prevent the persistent induction of neuroimmune signaling and the loss of cholinergic neurons in the adult basal forebrain following AIE. We further tested the hypothesis that blockade of neuroimmune signaling using the non-steroidal anti-inflammatory drug indomethacin during AIE would prevent the loss of basal forebrain cholinergic neurons. In the present study, we report that AIE treatment reduced ChAT+IR neurons in the adult female basal forebrain similar to male subjects. Adolescent intermittent ethanol exposure also decreased expression of the cholinergic neuron markers tropomyosin receptor kinase A (TrkA) and p75^NTR^, and increased phosphorylation of NF-κB p65 in the adult basal forebrain. Exercise and indomethacin treatment prevented that AIE-induced loss of cholinergic neuron markers and increased phosphorylation of NF-κB p65 in the basal forebrain. These novel findings are consistent with AIE causing lasting activation of the neuroimmune system that contributes to the persistent loss of basal forebrain cholinergic neurons in adulthood.

## Methods and materials

### Animals

Male and female Wistar rats bred and reared at the University of North Carolina at Chapel Hill were used in this study. On the day following birth (postnatal day [P]1), litters were culled to 10 pups with six males and four females retained when possible. Culled pups were euthanized by CO2 and decapitation as required by the Institutional Animal Care and Use Committee (IACUC) of the University of North Carolina at Chapel Hill (Protocol #: 17–253). Pups remained with their dams in standard clear plastic tubs with shavings until group housing with same sex littermates at the time of weaning on P21. All subjects were housed in a temperature- (20°C) and humidity-controlled vivarium on a 12 hr/12 hr light/dark cycle (light onset at 0700 hr), and provided *ad libitum* access to food and water. This study was carried out in strict accordance with the recommendations in the Guide for the Care and Use of Laboratory Animals of the National Institutes of Health. Animals are treated in an Assessment and Accreditation of Laboratory Animal Care (AAALAC) accredited facility. The protocol was approved by the Institutional Animal Care and Use Committee (IACUC) of the University of North Carolina at Chapel Hill (Protocol Number: 17–253). All efforts were made to minimize suffering. Any subject evidencing suffering or distress was euthanized according to our IACUC protocol.

### Adolescent intermittent ethanol (AIE) paradigm

On P21, Wistar rats were randomly assigned to either: (i) AIE (N = 119; Male: n = 111, Female: n = 8) or (ii) water control (CON; N = 103; Male: n = 95, Female: n = 8) conditions. To minimize the impact of litter variables, no more than one subject from a given litter was assigned to a single experimental condition. From P25 to P55, AIE subjects received a single daily intragastric (i.g.) administration of ethanol (5.0 g/kg, 20% ethanol, w/v) in the AM on a two-day on/two-day off schedule, and CON subjects received comparable volumes of water on an identical schedule. The body weight changes dramatically during rat adolescence (i.e., approximately 4-fold [~80 g– 400 g]), so we treat with g/kg to match body weight. In a separate study aimed at assessing the impact of adolescent age of binge ethanol exposure on basal forebrain ChAT+ neuron populations, male subjects (N = 32; CON: n = 8, early adolescence: n = 8, late adolescence: n = 8, complete AIE: n = 8) were treated with ethanol as described above from either P25 –P39 (early adolescence) or P40 –P55 (late adolescence) and tissue collected on P80 for comparison to subjects that received AIE treatment from P25 –P55. Tail blood was collected from AIE- and CON-treated subjects one hr after treatment on P38 and P54 to assess blood ethanol concentrations (BECs) using a GM7 Analyzer (Analox; London, UK. Body weights were assessed through AIE to the conclusion of experimentation.

### Voluntary wheel running exposure

To determine if voluntary exercise would prevent the AIE-induced loss of cholinergic neuron markers in adulthood, male CON- and AIE-treated animals (n = 8 per group) were pair-housed with non-litter mates on P24 in either standard cages (No exercise) or cages containing running wheels (Exercise). All subjects were observed daily for running wheel use and remained in their respective housing conditions 24 hr per day until sacrifice. All groups were housed in pairs as social isolation can counteract the beneficial effects of exercise [39, 40]. The exercise apparatus consisted of specially designed cages with a running wheel attachment (Panlab designed for Harvard Apparatus, Barcelona, Spain; Model LE904) connected to a LE3806 Multicounter (Panlab) allowing for collection of daily wheel revolutions. The wheel diameter was 36 cm with a lane width of 10 cm that was attached to a cage that measured 42 cm × 26 cm × 19 cm. All exercise-exposed subjects were observed using the running wheels and subject pairs ran a total average of 440 km that did not differ as a function of treatment condition (CON: 408 km [±39 km]; AIE: 473 km [±68 km]; one-way ANOVA: *p* > 0.4). Mean BECs (±SEM) were unaffected by running wheel exposure on either P38 (AIE/No exercise: 170 mg/dL [±11], AIE/Exercise: 158 mg/dL [±7]; one-way ANOVA: *p* > 0.3) or P54 (AIE/No exercise: 180 mg/dL [±12], AIE/Exercise: 184 mg/dL [±15]; one-way ANOVA: *p* > 0.8). Throughout the experiment, all subjects evidenced dramatic increases in body weight across age (main effect of Age: *p* < 0.01). While we did not observe an effect of exercise or treatment on body weight during AIE, we found that no exercise CON adult (P80) subjects weighed approximately 8% (~35 g) more than subjects in the other conditions. Subjects were sacrificed on P80 and brain tissue collected for assessment of cholinergic neuron markers.

### Indomethacin treatment

To determine if blockade of neuroinflammation would prevent the AIE-induced loss of basal forebrain cholinergic neuron markers, a separate group of CON- and AIE-treated male subjects (n = 8 per group) received treatment with either the non-steroidal anti-inflammatory drug indomethacin (4.0 mg/kg, i.p., Sigma-Aldrich, St. Louis, MO) or vehicle 30 min before AIE treatment on a two-day on/two-day off schedule. Indomethacin was suspended in 0.1 mL dimethyl sulfoxide and brought to a concentration of 0.8 mg/mL with sterile phosphate-buffered saline (pH: 7.4). Since our laboratory previously reported that AIE reduced ChAT+IR cholinergic neuron cell populations in the adolescent basal forebrain (i.e., P56) that persisted into adulthood [16], subjects were sacrificed 24 hr after the conclusion of AIE on P56 and tissue extracted for assessment of cholinergic neurons.

### Lipopolysaccharide (LPS) treatment

To mimic the persistent effects of neuroimmune activation by AIE, a separate group of CON- and AIE-treated male subjects (n = 8 per group) received either a single injection of LPS (1.0 mg/kg, i.p. in sterile 0.9% saline; E. Coli, serotype 0111:B4; Sigma-Aldrich) or a comparable volume of vehicle on P70. Subjects were sacrificed 10 days later on P80 and brain tissue collected for assessment of cholinergic neurons.

### Perfusion and tissue collection for immunohistochemistry

At the conclusion of each experiment, subjects were anesthetized with a euthanasia dose of sodium pentobarbital (100 mg/kg, i.p.) and transcardially perfused with 0.1 M phosphate-buffered saline (PBS, pH 7.4) followed by 4.0% paraformaldehyde in PBS. Brains were excised and post-fixed in 4.0% paraformaldehyde for 24 hr at 4°C followed by four days of fixation in a 30% sucrose solution. Coronal sections were cut (40 μm) on a sliding microtome (MICROM HM450; ThermoScientific, Austin, TX), and sections were sequentially collected into well plates and stored at -20°C in a cryoprotectant solution (30% glycol/30% ethylene glycol in PBS).

### Immunohistochemistry

Free-floating basal forebrain tissue samples (every 6^th^ section; approximately Bregma: 1.60 mm– 0.20 mm based on the atlas of Paxinos and Watson [41]) were washed in 0.1 M PBS, incubated in 0.6% H_2_O_2_ to inhibit endogenous peroxidases, and blocked with normal serum (MP Biomedicals, Solon, OH). Sections were incubated in a primary antibody solution containing blocking solution and either goat anti-choline acetyltransferase (ChAT; Millipore, Temecula, CA, Cat. #AB144P), rabbit anti-TrkA (Millipore, Cat. #06–574), mouse anti-p75^NTR^ (Millipore, Cat. #MAB365), or rabbit anti-phosphorylated NF-κB p65 Ser 536 (pNF-κB p65; Abcam, Cambridge, MA, Cat. #ab86299) for 24 hr at 4°C. Sections were washed with PBS, incubated for one hr in a biotinylated secondary antibody (Vector Laboratories, Burlingame, CA), and incubated for one hr in avidin-biotin complex solution (Vectastain ABC Kit; Vector Laboratories). The chromogen, nickel-enhanced diaminobenzidine (Sigma-Aldrich), was used to visualize immunoreactivity. Tissue was mounted onto slides, dehydrated, and cover slipped. Negative control for non-specific binding was conducted on separate sections employing the abovementioned procedures omitting the primary antibody.

### Microscopic quantification and image analysis

Across experiments, BioQuant Nova Advanced Image Analysis software (R&M Biometric, Nashville, TN) was used for image capture and quantification of immunohistochemistry. Representative images were captured using an Olympus BX50 microscope and Sony DXC-390 video camera linked to a computer. For each measure, the microscope, camera, and software were background corrected, and normalized to preset light levels to ensure fidelity of data acquisition.

Immunohistological assessment was performed in the medial septum and vertical limb of the diagonal band (Ch1/Ch2) as we previously discovered that AIE causes a persistent loss of ChAT+IR cells throughout the cholinergic nuclei of the brain [16]. A modified unbiased stereological quantification method was used to quantify ChAT-, TrkA-, p75^NTR^-, and pNF-κB p65-immunopositive cells in the rat basal forebrain. We previously reported that comparison of traditional unbiased stereological methodology with our modified unbiased stereological approach yielded nearly identical values relative to control subjects [42]. The outlined regions of interest were determined and data expressed as cells/mm^2^. Somal size was determined using BioQuant Nova Advanced Image Analysis software (R&M Biometric).

### Fluorescent immunohistochemistry and microscopy

To assess cholinergic neuron marker colocalization, free-floating basal forebrain sections were processed similar to previously reported methods [32, 33]. Briefly, sections collected from subjects sacrificed on P80 were washed in 0.1 M Tris-buffered saline (TBS), antigen retrieval performed by incubation in Citra solution (BioGenex, Fremont, CA) for one hr at 70°C, and blocked with normal horse serum (MP Biomedicals). Sections were incubated for 48 hr at 4°C in a primary antibody cocktail of goat anti-choline acetyltransferase (ChAT; Millipore) with an antibody against TrkA (rabbit anti-TrkA [Millipore]) and p75^NTR^ (mouse anti-p75^NTR^ [Millipore]). Sections were then washed in TBS and incubated for 2 hr at room temperature in the secondary antibody cocktail (rabbit Alexa Fluor 594, mouse Alexa Fluor 488, and goat Alexa Fluor 350; Invitrogen, Carlsbad, CA). Tissue was mounted onto slides and cover slipped using Prolong Gold Anti-Fade mounting media (Life Technologies, Grand Island, NY). Immunofluorescent images were obtained using a DS-RiZ scope (Nikon Inc., Melville, NY) and colocalization quantified using NIS Elements AR46 (Nikon Inc.).

### Tissue dissection, RNA extraction, and reverse transcription polymerase chain reaction (RTPCR)

On P80, male subjects were anesthetized with a euthanasia dose of sodium pentobarbital (100 mg/kg, i.p.) and transcardially perfused with 0.1 M phosphate-buffered saline (PBS, pH 7.4). Basal forebrain tissue from CON and AIE subjects, and hippocampal tissue from Exercise and No exercise CON and AIE subjects were dissected, rapidly frozen in liquid nitrogen, and stored at -80°C for mRNA extraction. Total mRNA was extracted from individual samples by homogenization in TRI reagent (Sigma-Aldrich) following the single-step method [43]. Total mRNA was reverse transcribed as previously described [33, 35]. SYBER green PCR Master Mix (Life Technologies) was used for the RTPCR. The real-time PCR was run with an initial activation for 10 min at 95°C, followed by 40 cycles of denaturation (95°C, 15 s), annealing/extension (57–58°C, 1 min), and finally a melt curve. The primer sequences are presented in Table 1. The threshold cycle (CT) of each target product was determined and the ΔΔCT method was used to calculate the percent change relative to CONs.

**Table 1:** List of primers for rat qPCR.

Primer	Forward	Reverse
*Chat*	5'-GCC CAA CCA AGC CAA GCA AT-3'	5'-AAA TGT CTT TGC GGG TGC CG-3'
*Trka*	5'-CCA TAT CAA GCG CCA GGA CA-3'	5'-GCA GTT TTG CAT CAG GTC CG-3'
*p75ntr*	5'-GCT GCT GAT TCT AGG GAT GTC-3'	5'-CAG TCT CCT CGT CCT GGT AGT-3'
*Ngf*	5'-CAT CGC TCT CCT TCA CAG AGT T-3'	5'-CAC GGG CAG CTA TTG GTT CA-3'
β*-actin*	5'-CTA CAA TGA GCT GCG TGT GGC-3'	5'-CAG GTC CAG ACG CAG GAT GGC-3'

### Statistical analysis

Statistical analysis was performed using SPSS (Chicago, IL). One-way analysis of variance (ANOVA) was used to assess BECs (Exercise: AIE/Exercise vs. AIE/No exercise), AIE cholinergic neuron marker loss (Treatment: CON vs. AIE), AIE age of exposure (Timing [CON vs. Early AIE vs. Late AIE vs. Complete AIE), and basal forebrain RTPCR data (Treatment). Individual 2 × 2 ANOVAs were used to assess data on body weights (Treatment × Exercise), sex differences (Treatment × Sex [Male vs. Female]), exercise immunohistochemistry (Treatment × Exercise), hippocampal RTPCR (Treatment × Exercise), indomethacin immunohistochemistry (Treatment × Indomethacin [Vehicle vs. Indomethacin]), and LPS immunohistochemistry (Treatment × LPS [Vehicle vs. LPS]). Post-hoc analyses were performed using Tukey’s HSD when significant interactions were observed. Significant interactions are discussed in relation to outcomes of Tukey’s HSD post-hoc tests. All values are reported as mean ± SEM, and significance was defined as *p* ≤ 0.05.

## Results

### Adolescent binge ethanol exposure reduces ChAT+IR neurons in the basal forebrain of adult male and female rats

**Fig 1:**
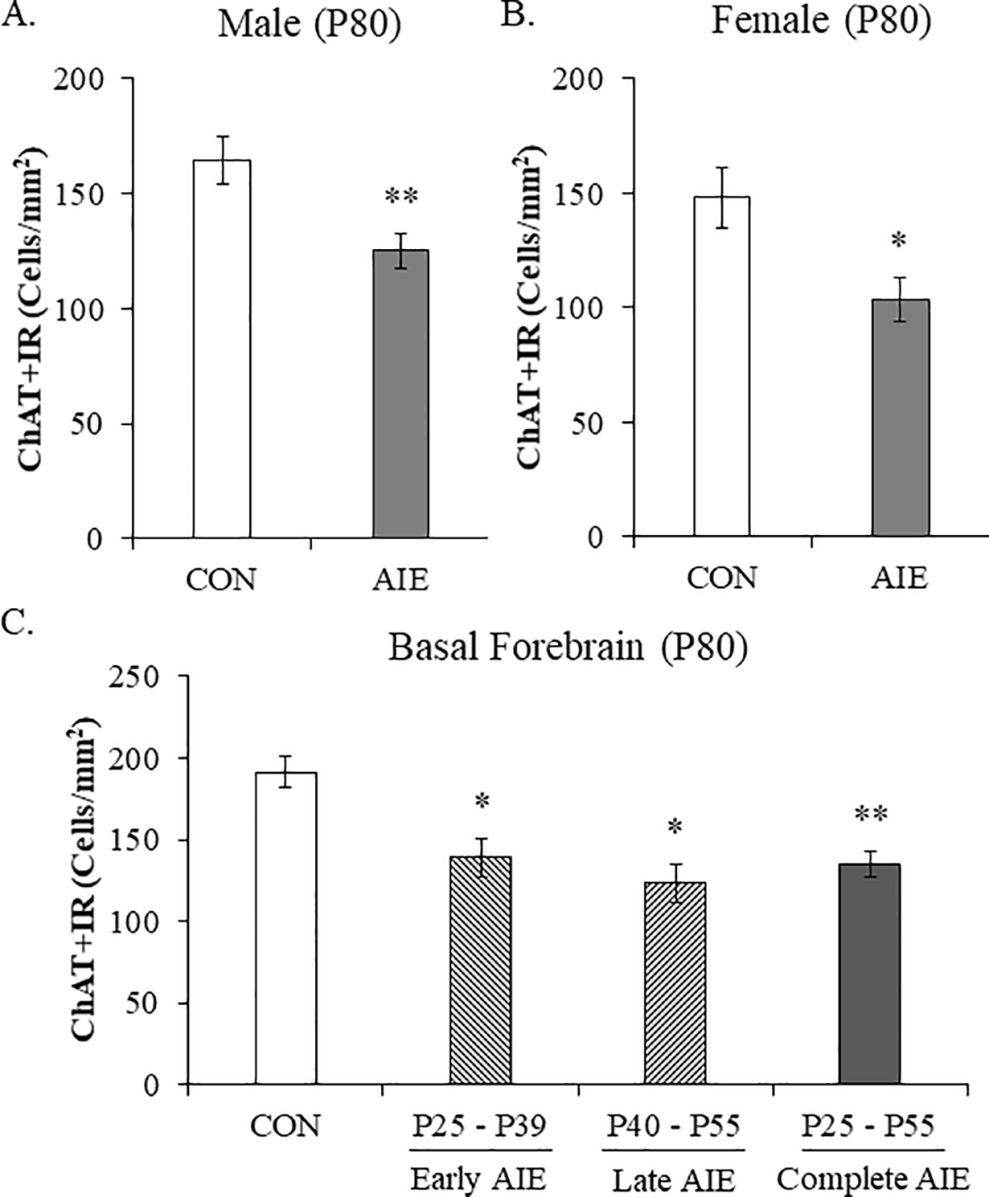
Adolescent intermittent ethanol (AIE) decreases choline acetyltransferase (ChAT)+IR cholinergic neurons in the adult male and female basal forebrain throughout adolescence. Modified unbiased stereological quantification of ChAT+IR neurons in the adult (P80) basal forebrain following AIE treatment revealed (A) a significant 27% (±5%) reduction in male subjects and (B) a significant 30% (±6%) reduction in female subjects, relative to CONs. Further, ChAT expression did not differ between male and female subjects. Given that we did not observe an effect of sex on ChAT+ expression, the remaining studies were conducted using male subjects. (C) Modified unbiased stereological quantification of ChAT+IR neurons following intermittent ethanol exposure during early adolescence (i.e. P25—P39) and late adolescence (i.e., P40 –P55) revealed a significant 27% (±6%) and 24% (±7%) reduction, respectively, in the adult male basal forebrain similar to the 29% (±4%) reduction observed following AIE treatment from P25 to P55. Data are presented as mean ± SEM. * *p* < 0.05, ** *p* < 0.01, relative to CONs.

Adolescent intermittent ethanol treatment has been found to reduce ChAT+IR neurons in the adolescent male basal forebrain (i.e., P55) that persists into adulthood (i.e., P220) [16–18]. However, to our knowledge, females have not been studied. We report here for the first time that AIE reduces ChAT+ neuron populations in the adult (i.e., P80) female basal forebrain similar to males (see Fig 1). Evaluation of ChAT+IR neurons revealed well defined heterogeneously distributed large and small darkly stained cell bodies and processes in CON- and AIE-treated female and male basal forebrain samples. Consistent with our previously published data, we found that AIE treatment led to a significant reduction of ChAT+IR neurons in the adult (P80) basal forebrain regardless of sex (main effect of Treatment: *F*_[1,27]_ = 16.2, *p* < 0.01). Interestingly, we did not observe a main effect of Sex or an interaction of Treatment × Sex indicating that there was not an effect of sex on cholinergic immunoreactivity (see Fig 1A and 1B). Since we did not observe a sex difference in ChAT expression, we collapsed the ChAT data and found that AIE led to a significant overall 26% (±4%) reduction of ChAT+IR neurons in the adult basal forebrain (one-way ANOVA: *F*_[1,30]_ = 15.0, *p* < 0.01). Given that we did not observe an effect of sex on cholinergic neuron marker expression, the remaining studies were conducted using male subjects. Thus, AIE treatment causes a comparable reduction of ChAT+IR neurons in the adult basal forebrain of males and females.

Emerging studies suggest that ethanol exposure might exert differential effects on the developing adolescent brain depending on the age of exposure [44]. Thus, we next assessed the impact of intermittent ethanol exposure during early adolescence (i.e., P25 –P39) and late adolescence (i.e., P40 –P55) relative to complete AIE exposure (i.e., P25 –P55) on ChAT+ neuron populations in the adult male basal forebrain. We found that both early (Tukey’s HSD: *p* < 0.05) and late adolescent (Tukey’s HSD: *p* < 0.05) intermittent ethanol exposure decreased ChAT+IR neuron populations similar to complete AIE treatment (Tukey’s HSD: *p* < 0.01) in the adult male basal forebrain, relative to CONs (see Fig 1C). These data reveal that intermittent ethanol exposure during early and late adolescence reduces the number of ChAT+IR cholinergic neurons in the adult basal forebrain similar to the loss observed in the complete AIE model.

### Adolescent intermittent ethanol reduces expression of multiple cholinergic neuron markers in the adult male basal forebrain

**Fig 2:**
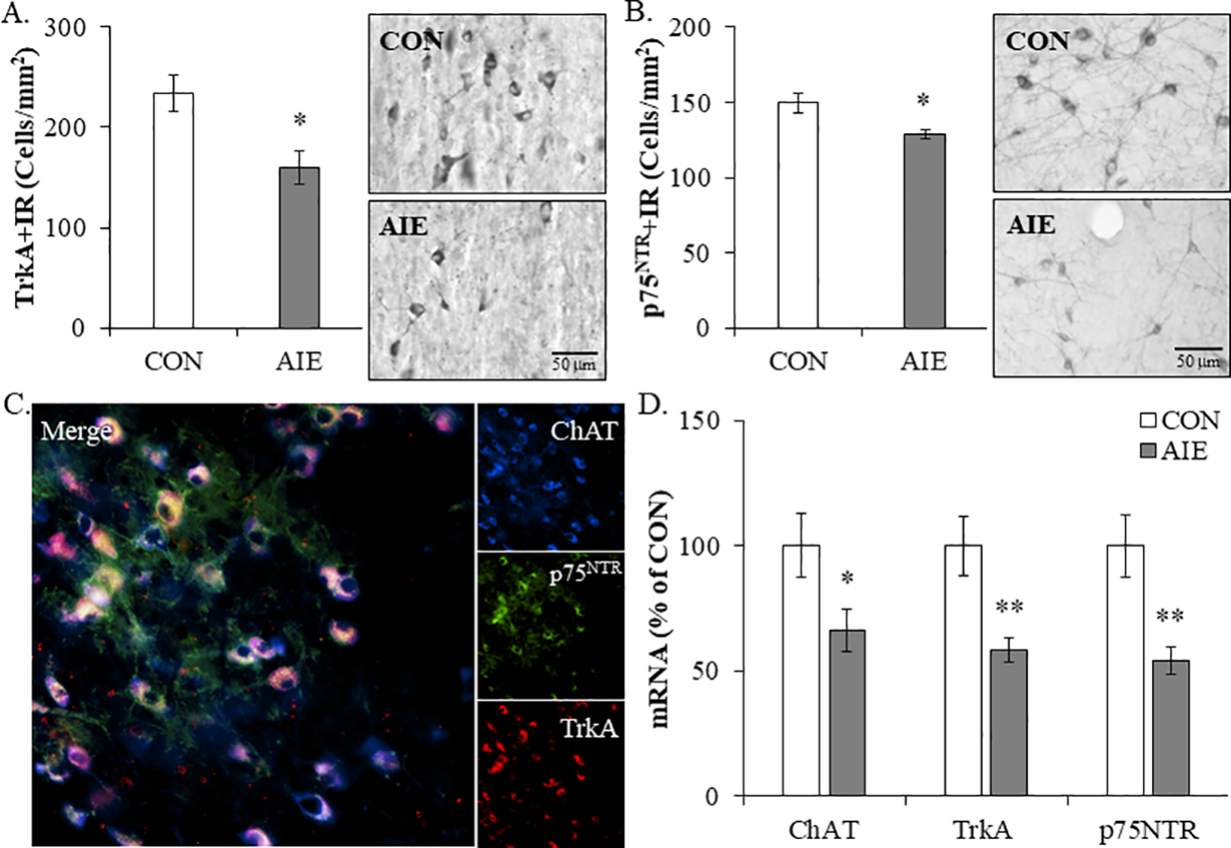
Adolescent intermittent ethanol (AIE) decreases expression of multiple cholinergic neuron markers in the adult male basal forebrain. (A) Modified unbiased stereological quantification of the high-affinity nerve growth factor (NGF) receptor tropomyosin receptor kinase A (TrkA) in the adult (P80) basal forebrain revealed a significant 32% (±8%) reduction in AIE-treated animals, relative to CONs. Representative photomicrographs of TrkA+IR cells in the adult basal forebrain of CON- and AIE-treated animals. (B) Modified unbiased stereological quantification of the low-affinity NGF receptor p75^NTR^ in the adult (P80) basal forebrain revealed a significant 14% (±2%) reduction in AIE-treated animals, relative to CONs. Representative photomicrographs of p75^NTR^+IR cells in the adult basal forebrain of CON- and AIE-treated animals. (C) Immunofluorescent co-labeling revealed a high degree of TrkA (red) and p75^NTR^ (green) colocalization with ChAT+IR neurons (blue) in the adult (P80) basal forebrain. (D) Quantitative PCR assessment of cholinergic neuron genes revealed a significant AIE-induced reduction of *ChAT* (34% [±9%]), *TrkA* (42% [±5%]), and *p75*^*NTR*^ (46% [±5%]) mRNA in adult (P80) basal forebrain tissue samples, relative to CONs. qPCR analyses were run in triplicate. Data are presented as mean ± SEM. * *p* < 0.05, ** *p* < 0.01, relative to CONs.

Since AIE led to the long-term reduction of ChAT+IR neurons, we next assessed the effect of AIE on expression of other known cholinergic neuron markers in the adult basal forebrain. Immunohistological assessment of the high-affinity nerve growth factor (NGF) receptor tropomyosin receptor kinase A (TrkA) and the low-affinity NGF receptor p75^NTR^, which are reported to be highly expressed in cholinergic neurons [45], revealed well defined darkly stained cell bodies and processes heterogeneously distributed throughout the basal forebrain of CON- and AIE-treated basal forebrain tissue samples (see Fig 2). We found that AIE treatment reduced TrkA+IR (one-way ANOVA: *F*_[1,12]_ = 7.5, *p* < 0.05; see Fig 2A) and p75^NTR^+IR (one-way ANOVA: *F*_[1,15]_ = 8.4, *p* < 0.05; see Fig 2B) cells in the adult basal forebrain, relative to CONs. Immunofluorescent co-labeling studies revealed a high degree of TrkA and p75^NTR^ colocalization with ChAT-immunoreactive cells in the basal forebrain (see Fig 2C). Measures of cholinergic neuron mRNA found a significant reduction of *ChAT* (one-way ANOVA: *F*_[1,12]_ = 4.9, *p* < 0.05), *TrkA* (one-way ANOVA: *F*_[1,12]_ = 10.7, *p* < 0.01), and *p75*^*NTR*^ (one-way ANOVA: *F*_[1,12]_ = 11.3, *p* < 0.01) in the basal forebrain of adult AIE-treated subjects, relative to CONs (see Fig 2D). Taken together, these data reveal that AIE treatment decreases ChAT, TrkA, and p75^NTR^, which is consistent with reduced cholinergic neurons in the adult basal forebrain following AIE.

### Phosphorylated NF-κB p65 immunoreactivity is increased in the basal forebrain of adult male AIE-treated rats

**Fig 3:**
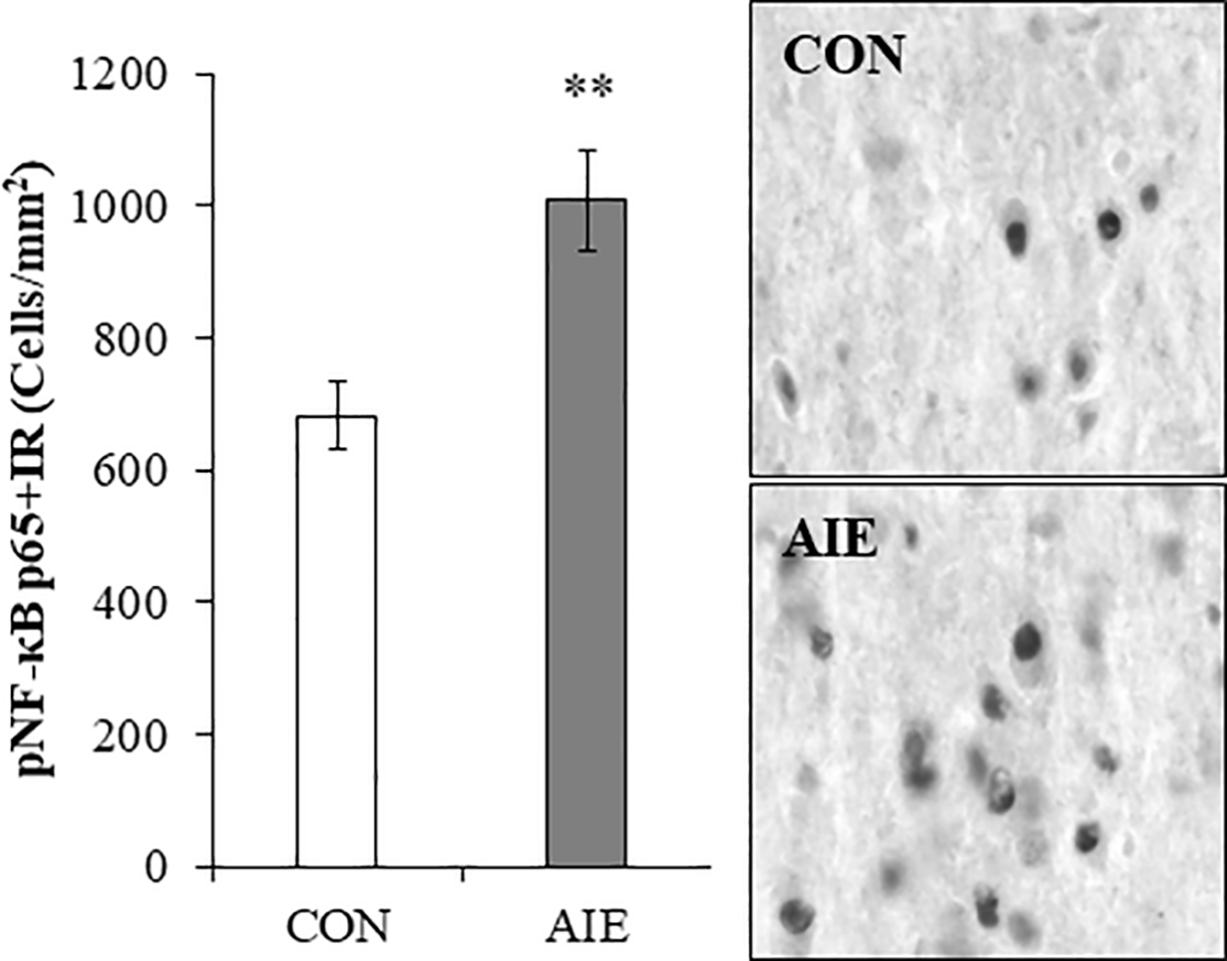
Increased expression of phosphorylated nuclear factor kappa-light-chain-enhancer of activated B cells p65 (pNF-κB p65) in the basal forebrain of adolescent intermittent ethanol (AIE)-treated adult male rats. Modified unbiased stereological quantification of pNF-κB p65+IR cells revealed a 48% (±11%) increase in the adult (P80) basal forebrain of AIE-treated animals, relative to CONs. Representative photomicrographs of pNF-κB p65+IR cells in the adult basal forebrain of CON- and AIE-treated animals. Data are presented as mean ± SEM. ** *p* < 0.01, relative to CONs.

Accumulating evidence reveals that AIE treatment causes a persistent upregulation of multiple neuroimmune signaling molecules throughout the adult brain [32, 33, 35, 46–48] and induction of neuroimmune signaling has been linked to ethanol-induced neurodegeneration [49, 50]. NF-κB is a transcription factor known to induce neuroimmune genes [36]. We assessed the transcriptionally active phosphorylated NF-κB p65 in the basal forebrain of adult rats (P80) following AIE treatment. Immunohistological assessment of pNF-κB p65+IR revealed darkly stained cell nuclei heterogeneously distributed throughout the basal forebrain. Assessment of pNF-κB p65+IR revealed a significant increase in the adult AIE-treated subjects, relative to CONs (one-way ANOVA: *F*_(1,13)_ = 11.6, *p* < 0.01; see Fig 3). Thus, AIE treatment results in a persistent long-term increase of pNF-κB p65+IR cells in the adult basal forebrain.

### Voluntary exercise blocks the AIE-induced cholinergic neuron pathology and increased phosphorylated NF-κB p65 in the adult male basal forebrain

**Fig 4:**
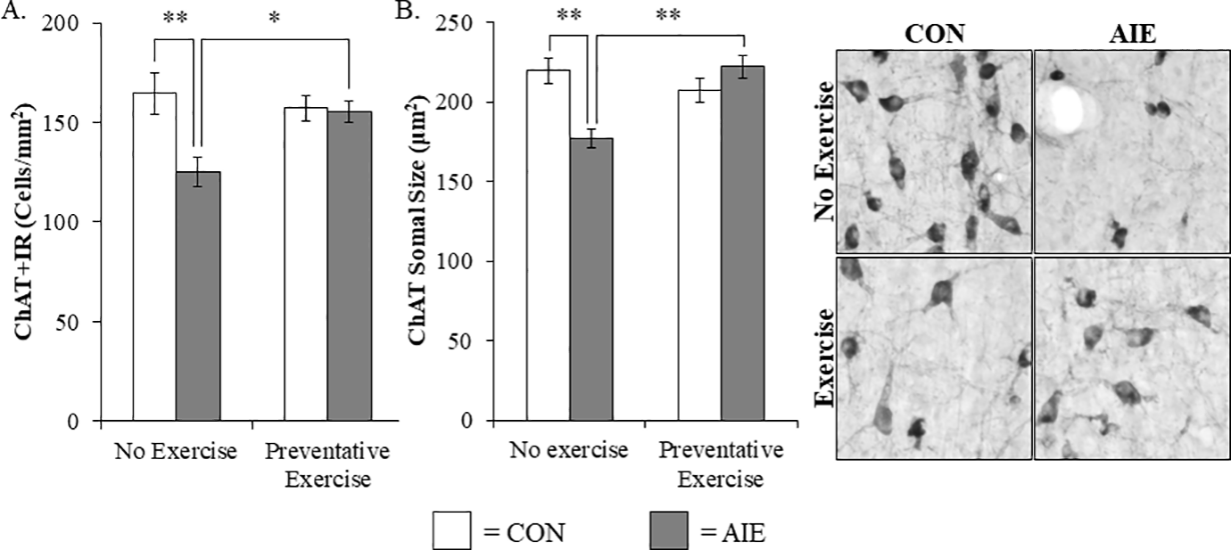
Wheel running prevents the adolescent intermittent ethanol (AIE)-induced loss and somal shrinkage of choline acetyltransferase (ChAT)+IR cholinergic neurons in the basal forebrain of adult male rats. (A) Modified unbiased stereological assessment revealed a 24% (±4%) reduction of ChAT+IR neurons in the adult (P80) basal forebrain of AIE-treated subjects, relative to CONs. Running wheel exposure from P24 to P80 did not affect ChAT expression in CONs, but did prevent the AIE-induced loss of ChAT+IR neurons, relative to the non-exercising AIE animals. (B) Analysis of ChAT+IR neuron somal size revealed a significant 19% (±3%) reduction in the adult basal forebrain of AIE-treated subjects, relative to CONs. Wheel running did not affect ChAT neuron somal size in CONs, but did prevent the AIE-induced ChAT+IR neuron somal shrinkage in the adult basal forebrain, relative to non-exercising AIE animals. Representative photomicrographs of ChAT+IR neurons in the adult basal forebrain from CON- and AIE-treated animals across exercise conditions. Data are presented as mean ± SEM. * *p* < 0.05, ** *p* < 0.01.

**Fig 5:**
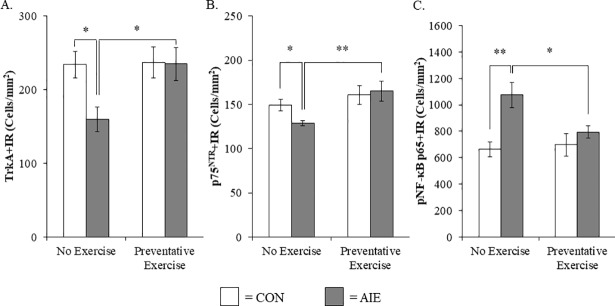
Voluntary exercise prevents the adolescent intermittent ethanol (AIE)-induced loss of tropomyosin receptor kinase A (TrkA)- and p75^NTR^-immunoreactive cells as well as the increased expression of phosphorylated nuclear factor kappa-light-chain-enhancer of activated B cells p65 (pNF-κB p65) in the adult male basal forebrain. (A) Modified unbiased stereological quantification of the high-affinity nerve growth factor (NGF) receptor TrkA in the adult (P80) basal forebrain revealed a significant 32% (±8%) reduction in AIE-treated animals, relative to CONs. Running wheel exposure from P24 to P80 did not affect TrkA expression in CONs, but did prevent the AIE-induced loss of TrkA+IR neurons, relative to the non-exercising AIE animals. (B) Modified unbiased stereological quantification of the low-affinity NGF receptor p75^NTR^ in the adult (P80) basal forebrain revealed a significant 14% (±2%) reduction in AIE-treated animals, relative to CONs. Wheel running alone did not affect p75^NTR^ expression in CONs, but did prevent the AIE-induced loss of p75^NTR^+IR neurons, relative to the non-exercising AIE animals. (C) Modified unbiased stereological quantification of pNF-κB p65+IR cells revealed a 62% (±14%) increase in the adult (P80) basal forebrain of AIE-treated animals, relative to CONs. While voluntary wheel running alone did not affect pNF-κB p65, it did prevent the AIE-induced increase of pNF-κB p65+IR cells, relative to the non-exercising AIE subjects. Data are presented as mean ± SEM. * *p* < 0.05, ** *p* < 0.01.

Previous studies have found that AIE increases adult neuroimmune signaling in the hippocampus and prefrontal cortex contributing to neurodegeneration [51]. Our finding of an AIE-induced increase of pNF-κB p65+IR in the basal forebrain is consistent with neuroimmune activation and/or other mechanisms contributing to the loss of ChAT+IR neurons. Previous studies have found that voluntary exercise can reverse ChAT+IR neurodegeneration in a rodent thiamine deficiency model [38] prompting an investigation of AIE combined with wheel running. We observed a reduction of ChAT+IR neurons in the basal forebrain of adult AIE-treated animals (Tukey’s HSD: *p* < 0.01), relative to CONs. While wheel running alone did not affect ChAT expression in CON subjects, it did prevent the AIE-induced loss of ChAT+IR neuron populations in the adult basal forebrain (Tukey’s HSD: *p* < 0.05; see Fig 4A). Assessment of ChAT+IR neuron somal size, which provides an index of cholinergic neuron health [52], revealed a significant reduction in the adult basal forebrain of AIE-treated subjects (Tukey’s HSD: *p* < 0.01), relative to CONs. Exposure to exercise did not affect somal size in the CONs, but prevented the AIE-induced ChAT+IR neuron somal shrinkage in the adult basal forebrain (Tukey’s HSD: *p* < 0.01; see Fig 4B). Assessment of TrkA+IR neuron populations revealed a significant reduction in the basal forebrain of adult AIE-treated animals (one-way ANOVA: *F*_(1,11)_ = 7.5, *p* < 0.05), relative to CONs. Wheel running alone did not affect TrkA+IR in CON subjects, but prevented the AIE-induced loss of TrkA+IR neuron populations in the adult basal forebrain (one-way ANOVA: *F*_(1,12)_ = 6.4, *p* < 0.05; see Fig 5A). Similarly, p75^NTR^+IR neuron populations were reduced in the basal forebrain of adult AIE-treated animals (one-way ANOVA: *F*_(1,14)_ = 8.4, *p* < 0.05), relative to CONs. Exposure to exercise did not affect p75^NTR^+IR in the CONs, but prevented the AIE-induced loss of p75^NTR^+IR neuron populations in the adult basal forebrain (one-way ANOVA: *F*_(1,14)_ = 9.6, *p* < 0.01; see Fig 5B). Interestingly, we also found that levels of *NGF* mRNA were reduced by 36% (±4%) in the hippocampus of adult AIE-treated animals (Tukey’s HSD: *p* < 0.01), relative to CONs. While wheel running alone did not affect *NGF* mRNA levels, AIE treatment combined with wheel running prevented the AIE-induced decrease of *NGF* mRNA (Tukey’s HSD: *p* < 0.05; data not shown). Exposure to AIE led to a significant increase of pNF-κB p65+IR cells in the adult basal forebrain (Tukey’s HSD: *p* < 0.01), relative to CONs. While exercise exposure did not affect expression of pNF-κB p65+IR in the CONs, it did prevent the AIE-induced increase of pNF-κB p65+IR cells in the adult basal forebrain (Tukey’s HSD: *p* < 0.05; see Fig 5C). Thus, wheel running prevented the AIE-induced increase of the neuroimmune marker pNF-κB p65+IR as well as the AIE-induced loss of cholinergic neuron markers in the adult basal forebrain.

### Treatment with the anti-inflammatory drug indomethacin prevents the AIE-induced loss of cholinergic neuron markers and increased phosphorylated NF-κB p65 in the male basal forebrain

**Fig 6:**
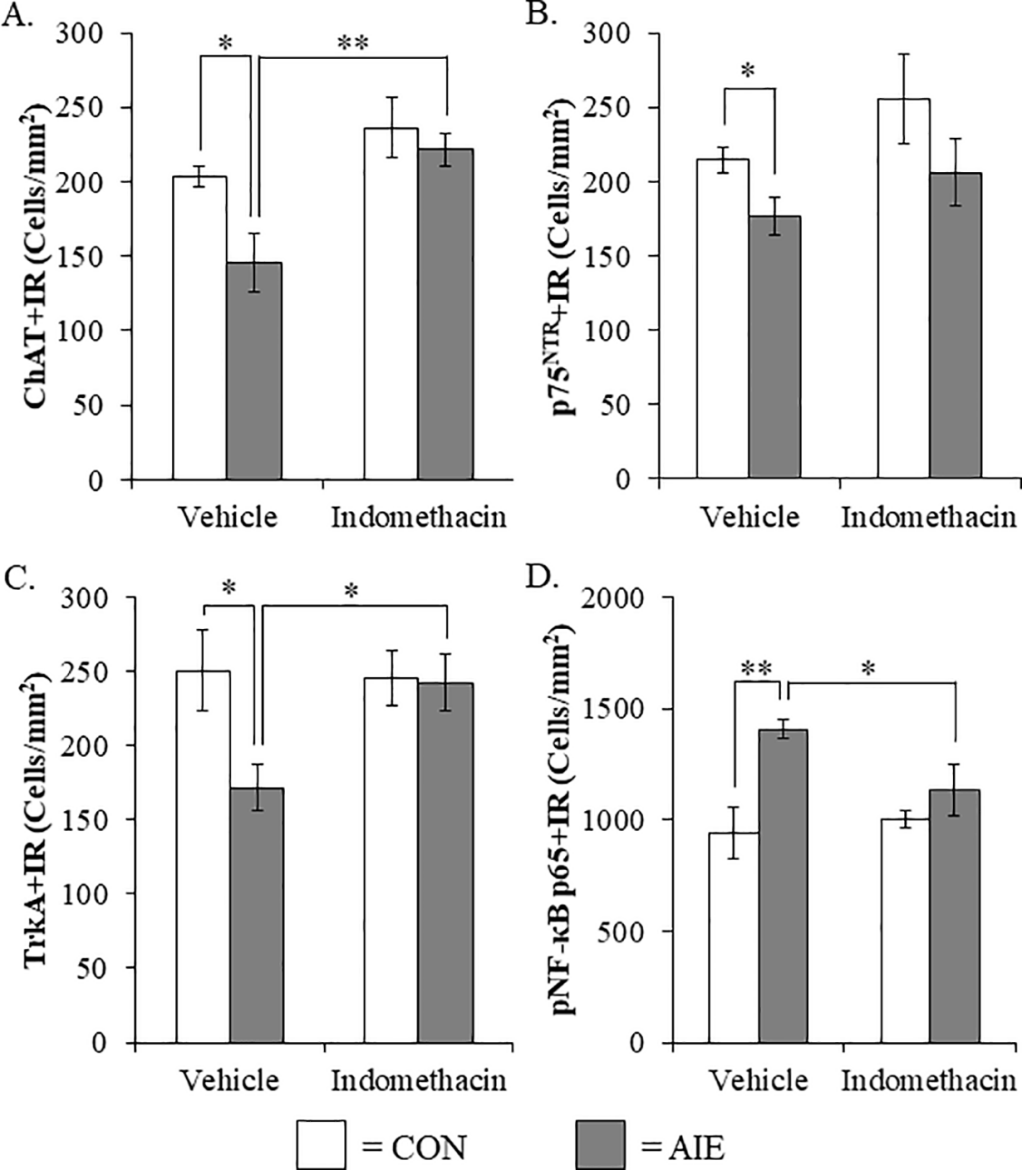
Indomethacin treatment prevented the adolescent intermittent ethanol (AIE)-induced loss of cholinergic neuron markers and increased phosphorylation of the proinflammatory transcription factor nuclear factor kappa-light-chain-enhancer of activated B cells p65 (phosphorylated [pNF-κB p65]) in the male basal forebrain. (A) Modified unbiased stereological assessment of choline acetyltransferase (ChAT)+IR neurons revealed a significant 28% (±10%) reduction in the basal forebrain of AIE-treated animals, relative to CONs. While indomethacin alone did not affect ChAT expression, it did prevent the AIE-induced loss of ChAT+IR neurons, relative to vehicle-treated AIE subjects. (B) Modified unbiased stereological assessment of the low-affinity nerve growth factor (NGF) receptor p75^NTR^ revealed a significant 18% (±6%) reduction in the AIE-treated animals, relative to CONs. Indomethacin treatment did not affect p75^NTR^ expression in CON- or AIE-treated subjects. (C) Modified unbiased stereological quantification of the high-affinity NGF receptor tropomyosin receptor kinase A (TrkA) in the adult (P80) basal forebrain revealed a significant 31% (±6%) reduction in AIE-treated animals, relative to CONs. Indomethacin treatment alone did not affect TrkA expression, but did prevent the AIE-induced loss of TrkA+IR neurons, relative to vehicle-treated AIE subjects. (D) Modified unbiased stereological quantification of pNF-κB p65+IR cells revealed a 50% (±4%) increase in the basal forebrain of AIE-treated animals, relative to CONs. While indomethacin alone did not affect pNF-κB p65, it did prevent the AIE-induced increase of pNF-κB p65+IR cells, relative to the vehicle-treated AIE subjects. Data are presented as mean ± SEM. * *p* < 0.05, ** *p* < 0.01.

Although exercise prevented the AIE-induced increase of pNF-κB p65 and cholinergic pathology, multiple mechanisms may be involved. To more directly test an inflammatory mechanism, we used the anti-inflammatory drug indomethacin, which is known to be effective in reducing adolescent ethanol-induced inflammatory gene induction, neurodegeneration, and behavioral deficits [30, 53]. We observed a reduction of ChAT+IR neurons in the basal forebrain of AIE-treated animals, relative to CONs (one-way ANOVA: *F*_(1,12)_ = 7.2, *p* < 0.05). While indomethacin alone had no effect on CONs, indomethacin treatment prevented the AIE-induced loss of ChAT+IR neurons (one-way ANOVA: *F*_(1,12)_ = 11.0, *p* < 0.01; see Fig 6A). We observed a statistically significant reduction of p75^NTR^+IR cells in the AIE-treated animals (one-way ANOVA: *F*_(1,10)_ = 6.2, *p* < 0.05) whereas indomethacin treatment did not affect the AIE-induced loss of p75^NTR^+IR cells (see Fig 6B). Assessment of TrkA+IR revealed a reduction in the AIE-treated basal forebrain, relative to CONs (one-way ANOVA: *F*_(1,10)_ = 6.2, *p* < 0.05). While indomethacin did not affect TrkA expression in CONs, indomethacin treatment prevented the AIE-induced loss of TrkA+IR cells in the basal forebrain (one-way ANOVA: *F*_(1,10)_ = 8.1, *p* < 0.05; see Fig 6C). Further, AIE led to an increase of pNF-κB p65+IR in the basal forebrain, relative to CONs (one-way ANOVA: *F*_(1,12)_ = 14.9, *p* < 0.01). While indomethacin did not affect pNF-κB p65 in the CONs, indomethacin treatment combined with AIE prevented the increase of pNF-κB p65+IR (one-way ANOVA: *F*_(1,12)_ = 5.0, *p* < 0.05; see Fig 6D). Taken together, the anti-inflammatory drug indomethacin blocked the AIE-induced loss of cholinergic neuron markers and concomitant increase of pNF-κB p65 supporting the hypothesis that neuroimmune inflammatory signaling induced by AIE contributes to the loss of basal forebrain cholinergic neurons.

### Lipopolysaccharide treatment mimics the AIE-induced loss of cholinergic neuron markers and potentiated phosphorylated NF-κB p65 expression in the adult male basal forebrain

**Fig 7:**
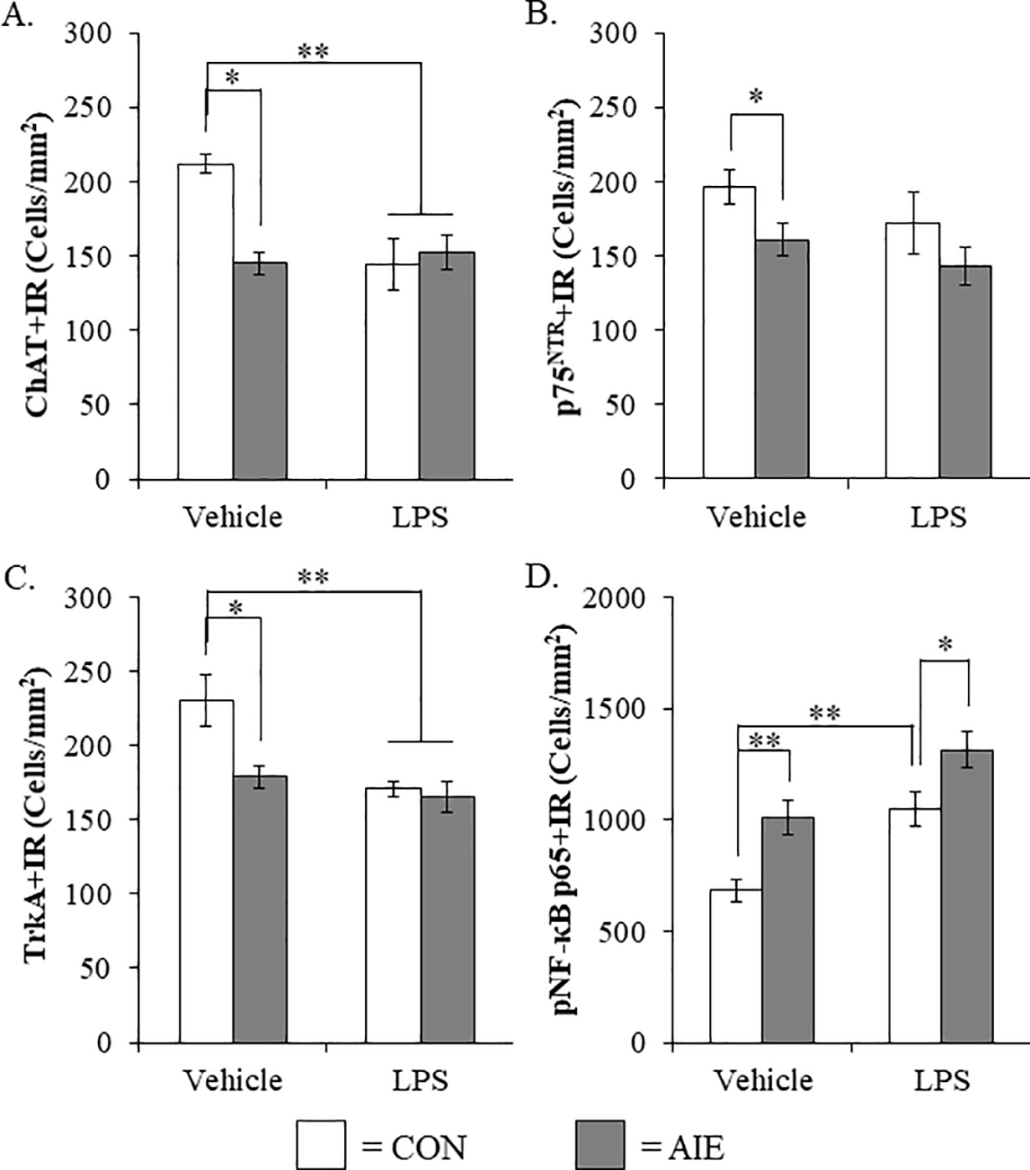
Lipopolysaccharide (LPS) treatment mimicked the adolescent intermittent ethanol (AIE)-induced loss of cholinergic neuron markers and increased phosphorylation of the proinflammatory transcription factor nuclear factor kappa-light-chain-enhancer of activated B cells p65 (phosphorylated [pNF-κB p65]) in the adult male basal forebrain. (A) Modified unbiased stereological assessment of choline acetyltransferase (ChAT)+IR neurons in the basal forebrain of adult (P80) rats revealed a significant reduction in AIE- (31% [±4]), CON+LPS- (32% [±8%]), and AIE+LPS-treated animals (28% [±5%]), relative to CONs. (B) Modified unbiased stereological assessment of p75^NTR^+IR neurons in the basal forebrain of adult (P80) rats revealed a significant reduction in AIE-treated animals (18% [±5]) while LPS treatment did not alter expression of p75^NTR^. (C) Modified unbiased stereological assessment of tropomyosin receptor kinase A (TrkA)+IR neurons in the basal forebrain of adult (P80) rats revealed a significant reduction in AIE- (22% [±5]), CON+LPS- (26% [±2]), and AIE+LPS-treated animals (28% [±5]), relative to CONs. (D) Modified unbiased stereological assessment of pNF-κB p65+IR cells in the basal forebrain of adult (P80) rats revealed a significant increase in AIE- (48% [±11%]), CON+LPS- (54% [±11%]), and AIE+LPS-treated animals (93% [±12%]), relative to CONs. Data are presented as mean ± SEM. * *p* < 0.05, ** *p* < 0.01.

To determine if neuroimmune activation recapitulates the AIE-induced loss of basal forebrain cholinergic neurons, a separate cohort of CON- and AIE-treated rats received a single dose of LPS (1.0 mg/kg i.p.) and were sacrificed on P80 similar to AIE animals. As in previous experiments in this study, AIE treatment reduced ChAT+IR (Tukey’s HSD: *p* < 0.01, see Fig 7A), p75^NTR^+IR (one-way ANOVA: *F*_(1,14)_ = 5.1, *p* < 0.05, see Fig 7B), and TrkA+IR (Tukey’s HSD: *p* < 0.05. see Fig 7C), relative to CONs. Lipopolysaccharide treatment reduced expression of ChAT in the LPS alone subjects (Tukey’s HSD: *p* < 0.01) and in the AIE+LPS subjects (Tukey’s HSD: *p* < 0.01), relative to CONs (see Fig 7A). Similarly, we found that LPS alone reduced TrkA+IR (Tukey’s HSD: *p* < 0.01) and AIE+LPS reduced TrkA+IR (Tukey’s HSD: *p* < 0.01), relative to CONs (see Fig 7C). As expected, pNF-κB p65 was increased in the AIE-treated animals, relative to CONs (one-way ANOVA: *F*_(1,13)_ = 11.6, *p* < 0.01). Lipopolysaccharide treatment increased pNF-κB p65+IR cells in CON- (one-way ANOVA: *F*_(1,13)_ = 14.6, *p* < 0.01) and AIE-treated (one-way ANOVA: *F*_(1,12)_ = 40.8, *p* < 0.01) animals respectively, relative to CONs (see Fig 7D). Interestingly, we observed higher levels pNF-κB p65+IR cells in the basal forebrain of AIE-treated animals exposed to LPS, relative to LPS-treated CONs (one-way ANOVA: *F*_(1,13)_ = 5.6, *p* < 0.05). Taken together, these data support our hypothesis that the inflammagen LPS mimics the AIE loss of cholinergic neuron markers and induction of basal forebrain neuroimmune signaling. Overall, these studies are consistent with AIE-induced neuroimmune signaling contributing to the loss of basal forebrain cholinergic neuron markers.

## Discussion

**Fig 8:**
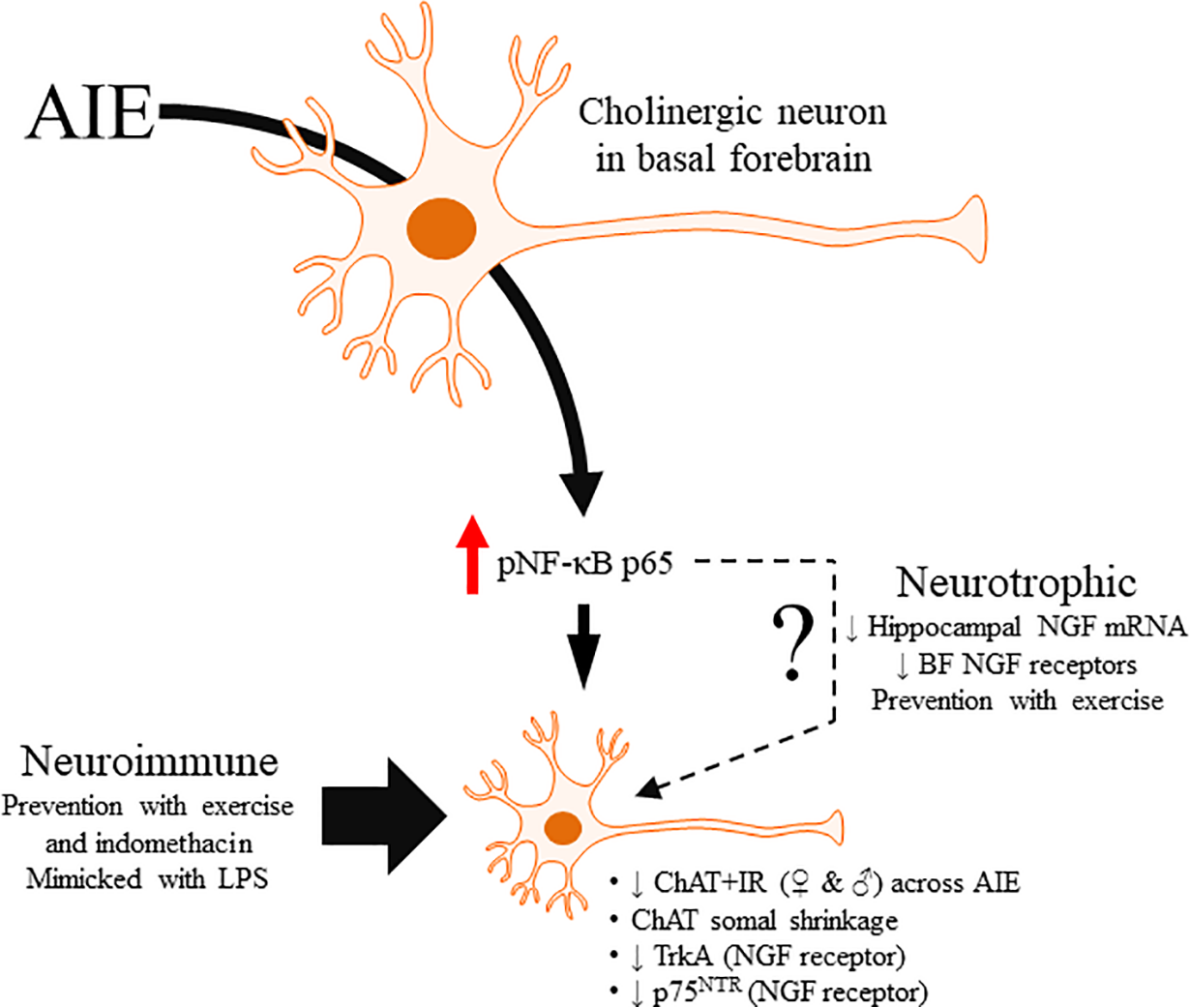
Simplified schematic depicting the proposed mechanism underlying the adolescent intermittent ethanol (AIE)-induced loss of basal forebrain cholinergic neurons. Adolescent binge ethanol exposure increases phosphorylation of the proinflammatory transcription factor nuclear factor kappa-light-chain-enhancer of activated B cells p65 (phosphorylated [pNF-κB p65]). This in turn results in the induction of neuroimmune signaling molecules that activates positive loops of amplification that persist into adulthood [54]. The present study suggests that this persistent proinflammatory neuroimmune activation contributes to the loss of basal forebrain cholinergic neurons that continues into adulthood. Indeed, exposure to either voluntary wheel running, which conveys immune modulatory effects, or treatment with the anti-inflammatory drug indomethacin prevents the loss of basal forebrain cholinergic neurons by preventing the AIE-induced increase of pNF-κB p65. Moreover, treatment with the endotoxin lipopolysaccharide (LPS) mimicked the AIE-induced loss of cholinergic neurons while increasing expression of pNF-κB p65. While these data implicate the neuroimmune system in the persistent AIE-induced loss basal forebrain cholinergic neurons, the precise mechanism remains to be fully elucidated. Interestingly, LPS treatment has previously been found to decrease hippocampal expression of the neurotrophin nerve growth factor (NGF) [55, 56], which is critical for basal forebrain cholinergic maintenance and survival [10]. Thus, AIE-induced neuroimmune activation may disrupt NGF inputs to the basal forebrain culminating in the persistent loss of cholinergic neurons. Thus, these data provide support for a neuroimmune mechanism underlying the AIE-induced loss of basal forebrain cholinergic neurons.

In the present study, we extend our research on the persistent AIE-induced reduction of ChAT+IR cholinergic neurons in the male basal forebrain with the discovery of (1) a loss of ChAT+ cholinergic neurons in AIE-treated adult female subjects, (2) a reduction of additional cholinergic neuron markers following AIE treatment, and (3) neuroimmune involvement in the AIE-induced loss of cholinergic neurons. We report here for the first time that AIE treatment, which models human adolescent binge drinking, led to a reduction of ChAT+IR neurons in the basal forebrain of adult female subjects similar to male AIE-treated rats. We also report that both early (i.e., P25 –P39) and late (i.e., P40 –P55) adolescent intermittent ethanol treatment similarly reduces ChAT+IR neurons. We also discovered that AIE treatment decreased expression of the high-affinity nerve growth factor (NGF) receptor tropomyosin receptor kinase A (TrkA) and the low-affinity NGF receptor p75^NTR^, both of which are markers of cholinergic neurons, in the adult basal forebrain. Further, we observed increased expression of pNF-κB p65+IR in the adult basal forebrain, which is a neuroimmune marker consistent with previous studies finding increased neuroimmune activation following AIE treatment [30]. We report here for the first time that exposure to wheel running from P24 –P80 prevented the AIE-induced loss of cholinergic neuron markers (i.e., ChAT, TrkA, and p75^NTR^) as well as the increased expression of pNF-κB p65 in the adult basal forebrain. These observations are consistent with previous studies finding that exercise blunts induction of proinflammatory cytokines in the basal forebrain following middle cerebral artery occlusion [37]. Further, the anti-inflammatory drug indomethacin blocked the AIE-induced loss of cholinergic neuron markers (i.e., ChAT and TrkA) as well as the increased expression of pNF-κB p65 in the basal forebrain. Previous studies have found that indomethacin blocks adolescent ethanol-induced neuroimmune induction and neurodegeneration in the hippocampus and cortex [30, 53]. In addition, the inflammagen LPS mimicked the AIE-induced loss of cholinergic neuron markers (i.e., ChAT and TrkA) and increased pNF-κB p65+IR in the adult basal forebrain. Taken together, these data support the hypothesis that adolescent binge ethanol-induced neuroimmune signaling contributes to the persistent loss of cholinergic neuron markers in the adult basal forebrain (see Fig 8).

Historically, AIE has been shown to reduce populations of ChAT+IR neurons in the basal forebrain of adult male rodents [16–18, 57], but to date no studies have assessed the effect of AIE on ChAT expression in the basal forebrain of adult females. In the present study, we found that expression of ChAT in control male and female Wistar rats did not differ as a function of sex, which is consistent with data reporting similar numbers of basal forebrain ChAT+IR neurons in aging male and female rats (i.e., 12 mo—25 mo; [58]). Importantly, AIE treatment reduced populations of ChAT+IR cholinergic neurons in the basal forebrain of adult female rats that was comparable to the loss observed in the adult male subjects. Although our other ChAT studies were conducted using male subjects, the findings of the current study indicate that the AIE-induced loss of ChAT is not sex dependent. Further, emerging studies suggest that ethanol exposure may exert differential effects on the developing adolescent brain depending on age of exposure [44]. However, we observed a comparable loss of ChAT+IR neurons in the basal forebrain of adult rats exposed to either early or late adolescent intermittent ethanol, relative to complete AIE. Together, these data reveal that AIE treatment causes a similar loss of ChAT+IR in the basal forebrain of male and female subjects. Further, intermittent ethanol exposure at different time points during adolescence caused a comparable reduction of ChAT+IR neurons supporting the observation that adolescence is a period of increased vulnerability of basal forebrain cholinergic neurons to the deleterious effects of ethanol exposure.

In addition to expressing the acetylcholine-synthesizing enzyme ChAT, basal forebrain ChAT+IR cholinergic neurons have been reported to co-express the high-affinity NGF receptor TrkA and the low-affinity NGF receptor p75^NTR^ [45, 59, 60]. Nerve growth factor, which undergoes retrograde transport along cholinergic axon projections from the cortex and hippocampus, regulates activity and survival of basal forebrain cholinergic neurons through its interactions with TrkA and p75^NTR^ located on cholinergic neurons [10, 61]. Consistent with the literature, we found that the majority of basal forebrain ChAT+IR cholinergic neurons co-expressed TrkA and p75^NTR^. Importantly, we report here for the first time that AIE treatment caused a persistent reduction of TrkA and p75^NTR^ protein and mRNA expression in the adult basal forebrain that paralleled the loss of ChAT. Diminished expression of cholinergic markers has also been reported in the post-mortem basal forebrain of human patients with Alzheimer’s disease [62, 63] and alcoholism [16]. Similar to our observations, reduced levels of ChAT, TrkA, and p75^NTR^ were reported in the basal forebrain of adult rats following 28 weeks, but not shorter intervals, of sole source of fluid (i.e., water or ethanol) [21, 64]. This observation, coupled with our finding that an identical intermittent ethanol treatment in adulthood did not affect basal forebrain cholinergic neuron populations following a 30-day period of abstinence [16], highlights the increased vulnerability of adolescent basal forebrain cholinergic neurons to the degenerative effects of ethanol.

Exercise prevents the AIE-induced loss of hippocampal neurogenesis [30], but the effects of exercise on the AIE-induced loss of cholinergic neurons has not yet been assessed. We report here for the first time that AIE combined with voluntary wheel running (i.e., P24 –P80) prevented the loss of cholinergic neuron markers (i.e., ChAT, TrkA, and p75^NTR^) in the adult basal forebrain. In line with our observations, Hall and Savage [38] reported that wheel running restored the loss of ChAT+IR neurons in a rodent model of thiamine deficiency-induced neurodegeneration. In addition to reducing cholinergic neuron marker expression in the adult basal forebrain, we found that AIE decreased somal size of the remaining ChAT+IR cholinergic neurons in the adult basal forebrain, which was prevented by wheel running in the AIE-treated animals. Similarly, fimbria/fornix transection and thiamine deficiency has been reported to reduce the somal size of surviving ChAT+IR neurons [38, 65]. Further, Nagahara and colleagues [66] found a decline in the size of cholinergic neurons (i.e., ~16%) in the basal forebrain of aged (24 years old) monkeys. Consistent with our results, Hall and Savage [38] found that wheel running reversed the somal shrinkage observed in the basal forebrain of thiamine deficient rats. These data on cholinergic somal size, which provides a general index of cholinergic neuron health [52], suggest that the cholinergic neurons that remain following AIE may be impaired necessitating further study to evaluate the function of the remaining cholinergic neurons. We also found that wheel running prevented the AIE-induced increase of pNF-κB p65 in the adult basal forebrain. Consistent with the anti-inflammatory effects of exercise, Ang and colleagues [37] found that 12 weeks of treadmill running blunted upregulation of *TNFα*, *IL-6*, and *IFN-γ* mRNA expression in the basal forebrain of rats exposed to middle cerebral artery occlusion. Taken together, these data reveal that voluntary exercise prevents the AIE-induced loss of cholinergic neuron markers and supports the hypothesis of a neuroimmune mechanism underlying the persistent loss of basal forebrain cholinergic neurons.

Although supportive of a neuroimmune mechanism underlying the AIE-induced loss of basal forebrain cholinergic neurons, exercise confers additional beneficial effects to the CNS (e.g., increased NGF and cerebral blood flow) that may be independent or a primary mechanism responsible for the prevention of neuroimmune signaling and the concomitant loss of cholinergic neurons. Indeed, in the present study we found that hippocampal *NGF* mRNA levels were reduced by AIE treatment and restored in the exercising AIE-treated animals. Similar to our findings, forced exercise has been reported to increase NGF levels in the hippocampus of adult rats [67]. The combined tropic and anti-inflammatory actions of exercise confound interpretation of the mechanism underlying the AIE-induced loss of cholinergic neurons. Thus, we next administered the non-steroidal anti-inflammatory drug indomethacin, which inhibits NF-κB activity [68], during AIE treatment and assessed cholinergic neuron marker expression in the basal forebrain. Indomethacin treatment during AIE blocked the increase in pNF-κB p65+IR and loss of cholinergic neuron markers (i.e., ChAT and TrkA) at P56 following the conclusion of AIE treatment further linking neuroimmune induction to cholinergic neuron loss. Additional studies are needed to determine if treatment after AIE could reverse inflammation and/or ChAT+ neuron loss. Previous studies have shown that AIE increases mRNA for HMGB1, multiple TLRs, MCP-1, TNFα, and other neuroimmune signals [32–35]. We used indomethacin, a known anti-inflammatory drug, and pNF-κB p65 as a marker of neuroimmune activation. Although indomethacin blocked increased pNF-κB p65 and the loss of cholinergic neuron markers, we do not know if all of the neuroimmune genes found to be induced by AIE are reduced by indomethacin or exercise. In a previous study, we found that exercise prevented the AIE-induced loss of hippocampal neurogenesis as well as the increase of pNF-κB p65 and induction of *HMGB1*, *TLR4*, *MCP*-1, *TNFα*, and *IκBα* genes [34] consistent with pNF-κB p65 being a reasonable marker for neuroimmune signaling. Our data extends previous studies reporting indomethacin blockade of adolescent ethanol-induced neuroimmune induction and neurodegeneration [30, 53]. In the present study, we also found that activation of the neuroimmune system using the proinflammatory immune activator LPS caused a loss of ChAT- and TrkA-immunopositive cholinergic neurons while increasing pNF-κB p65 expression, an active form of the pan-neuroimmune transcription factor, in the basal forebrain that mimicked the effects of AIE. Although we did observe LPS following AIE further increase pNF-κB p65+IR, we did not find an increase in the AIE-induced loss of cholinergic neuron markers. This could be due to different populations of ChAT+ neurons with varied vulnerability or a limited ability of neuroimmune signaling to reduce ChAT+ [9, 69]. Similar to our findings, LPS has been shown to reduce cholinergic neuron marker expression in *in vivo* and *in vitro* basal forebrain neuron cultures [23, 24]. Taken together, these data indicate that anti-inflammatory drug treatment blocks and neuroimmune system activation mimics the AIE-induced loss of basal forebrain cholinergic neurons providing support for the neuroimmune hypothesis of cholinergic neuron loss in the AIE model.

While not the focus of the current study, basal forebrain cholinergic neurons innervate the cortex, hippocampus, and other brain regions, and are consequently critical for cognitive function [2, 70]. The basal forebrain continues to undergo maturational refinement through adolescence [9] increasing its vulnerability to the neurotoxic effects of ethanol [71], and the AIE-induced loss of cholinergic neurons likely contributes to the neurocognitive deficits observed in adulthood [18, 32, 72]. The present data suggest that interventions aimed at preventing the loss of basal forebrain cholinergic neurons, such as exercise and indomethacin, might recover the observed cognitive dysfunction in adulthood following AIE. Indeed, indomethacin has been reported to restore ethanol-induced cognitive dysfunction in mice [53]. Consistent with the AIE model, a loss of basal forebrain cholinergic neuron markers is observed in human neurodegenerative disorders and alcoholism [16, 28], which likely contribute to the cognitive dysfunction that accompany these disorders. While the effects of exercise on human basal forebrain cholinergic neurons is unknown, exercise training as been shown to prevent age-related cognitive decline in humans [73] consistent with a beneficial effect of exercise on cholinergic neurons of the basal forebrain.

In conclusion, this is the first time sex differences in AIE-induced loss of ChAT+ neurons was reported in the adult basal forebrain. Our data also support the hypothesis that AIE-induced upregulation of neuroimmune signaling contributes to the persistent loss of basal forebrain cholinergic neuron markers in the adult basal forebrain. Adolescent intermittent ethanol exposure led to persistently increased phosphorylation of the proinflammatory nuclear transcription factor NF-κB p65 in the adult basal forebrain. Upregulation of NF-κB p65 leads to the induction of proinflammatory cytokines, oxidases, and other neuroimmune signaling molecules that lead to further NF-κB activation following cessation of ethanol exposure lending support to the establishment of positive loops of neuroimmune signaling in the AIE model that persists into adulthood [51]. Interventions aimed at inhibition of the increased phosphorylation of NF-κB p65, such as exercise or administration of the anti-inflammatory drug indomethacin prevents the AIE-induced loss of basal forebrain cholinergic neuron markers. Conversely, administration of neuroimmune activator LPS mimicked the AIE-induced increase of pNF-κB p65 and loss of cholinergic neuron markers in the adult basal forebrain. Together, these novel findings implicate proinflammatory pNF-κB p65-neuroimmune signaling in the AIE-induced loss of basal forebrain cholinergic neurons.
